# Motors and Motoring

**Published:** 1903-08-15

**Authors:** 


### Motors and Motoring.

The passage of the Motor Car Bill through the House of Commons may be taken to secure its adoption during the present session of Parliament. If this expectation be fulfilled, and the makers and users of motor cars placed, by its fulfilment, in a definite and recognisable position, by which both the enterprises of the former and the conduct of the latter may be more or less effectively regulated, we may reasonably expect to witness a considerable development of the industry, and a greatly increased employment of motor vehicles within the limits which the legislature will have laid down for their control. In these circumstances, it is difficult not to feel some anxiety with regard to the possible, or even probable, effects of motor-driving upon the somewhat unstable conditions of the nervous system which are only too frequently encountered in the present day, and which it is customary, and probably correct, to attribute to the stress and wear of civilisation and its requirements. The difficulties arising from meeting or passing horses are plainly temporary, for in the neighbourhood of London horses have ceased to take more notice of a car than they would of an omnibus, and the same effect of custom and education must inevitably soon be produced in all parts of the country. But, notwithstanding this, and even within the limits of speed laid down by the Committee of the House of Commons, the strain upon the driver is very severe, if only by reason of its continuous and unrelaxing character, and it may well be doubted whether it will not prove injurious or destructive to some, at least, of those who undergo it. The frequent little spurts which must be employed, if speed is to be maintained in a busy thoroughfare, in order to avoid detention between meeting vehicles, and to dart past a preceding one on its off-side with time and space to turn in towards the off-side of an approaching one at the next moment, together with the necessity for constant preparedness against the possible eccentricities of other drivers, these are conditions which demand a continuous alertness of a very exhausting character, and which would be likely before long, to lead to a very large elimination of the unfit from among those by whom they were undertaken. Such an elimination, unfortunately, would in most instances imply complete and permanent breakdown; and occasionally, at least, would be emphasised by more or less fatal or destructive accidents.

The common type of furious motorist, by whose misdeeds somewhat stringent legislation has been rendered necessary, may probably be regarded as a merely temporary phenomenon, a natural result of the moral and physical decadence which is common in the second generation of the newly rich. Young men, cursed with more money than they have either the wisdom or the virtue to use well, who have discarded the obsequiousness natural to their station without acquiring either courtesy or consideration for the rights of others, and whose nervous systems, often shaken by debauchery, are saturated with alcohol and tobacco, have been let loose to career along the roads without restraint. Any fate which may befall them will be regarded with much tranquillity by persons of better regulated minds; and society may reasonably be called upon to assist the law in keeping them within the bounds of propriety. With such we have nothing to do, our object being but to urge the necessity for prudence even in the proper and wholesome use of car driving. It is only necessary to watch the face of a driver in a busy road in order to realise the amount of strain which is thrown upon him. The tense muscles are a sufficient index of the corresponding tension of the nervous system generally; and no physiologist can doubt that such tension cannot be prudently endured for any long period of time, at least until it has been rendered second nature by experience or even by descent. There are, of course, many compensations, such as the exhilaration of rapid movement, the abundant supply of fresh air to the lungs, and the pleasure of exerting a new accomplishment. All these things may disguise the effort and may even render it more easy of performance, but they do not alter the essential conditions of the case.

